# Effect of antibiotic treatment and gamma-irradiation on cuticular hydrocarbon profiles and mate choice in tsetse flies (*Glossina m. morsitans*)

**DOI:** 10.1186/s12866-018-1292-7

**Published:** 2018-11-23

**Authors:** Tobias Engl, Veronika Michalkova, Brian L. Weiss, Güler D. Uzel, Peter Takac, Wolfgang J. Miller, Adly M. M. Abd-Alla, Serap Aksoy, Martin Kaltenpoth

**Affiliations:** 1 Insect Symbiosis Research Group, Max Planck Institute for Chemical Ecology, Jena, Germany; 2 Department for Evolutionary Ecology, Institute for Organismic and Molecular Evolution, Johannes Gutenberg-University Mainz, Mainz, Germany; 3 Department of Epidemiology of Microbial Diseases, Yale School of Public Health, New Haven, CT USA; 4 Institute of Zoology, Slovak Academy of Sciences, Bratislava, Slovakia; 5 Present Address: Department of Biological Sciences, Florida International University, Miami, FL USA; 6 Insect Pest Control Laboratory, Joint FAO/IAEA Division of Nuclear Techniques in Food & Agriculture, Vienna, Austria; 7 Institute of Chemical, Environmental and Biological Engineering, Research Area Biochemical Technology, Vienna University of Technology, Vienna, Austria; 8 Laboratories of Genome Dynamics, Department Cell and Developmental Biology, Medical University of Vienna, Vienna, Austria

**Keywords:** *Glossina morsitans*, Tsetse, Endosymbiont, *Wigglesworthia*, Cuticular hydrocarbons, Mate choice

## Abstract

**Background:**

Symbiotic microbes represent a driving force of evolutionary innovation by conferring novel ecological traits to their hosts. Many insects are associated with microbial symbionts that contribute to their host’s nutrition, digestion, detoxification, reproduction, immune homeostasis, and defense. In addition, recent studies suggest a microbial involvement in chemical communication and mating behavior, which can ultimately impact reproductive isolation and, hence, speciation. Here we investigated whether a disruption of the microbiota through antibiotic treatment or irradiation affects cuticular hydrocarbon profiles, and possibly mate choice behavior in the tsetse fly, *Glossina morsitans morsitans*. Four independent experiments that differentially knock down the multiple bacterial symbionts of tsetse flies were conducted by subjecting tsetse flies to ampicillin, tetracycline, or gamma-irradiation and analyzing their cuticular hydrocarbon profiles in comparison to untreated controls by gas chromatography – mass spectrometry. In two of the antibiotic experiments, flies were mass-reared, while individual rearing was done for the third experiment to avoid possible chemical cross-contamination between individual flies.

**Results:**

All three antibiotic experiments yielded significant effects of antibiotic treatment (particularly tetracycline) on cuticular hydrocarbon profiles in both female and male *G. m. morsitans*, while irradiation itself had no effect on the CHC profiles. Importantly, tetracycline treatment reduced relative amounts of 15,19,23-trimethyl-heptatriacontane, a known compound of the female contact sex pheromone, in two of the three experiments, suggesting a possible implication of microbiota disturbance on mate choice decisions. Concordantly, both female and male flies preferred non-treated over tetracycline-treated flies in direct choice assays.

**Conclusions:**

While we cannot exclude the possibility that antibiotic treatment had a directly detrimental effect on fly vigor as we are unable to recolonize antibiotic treated flies with individual symbiont taxa, our results are consistent with an effect of the microbiota, particularly the obligate nutritional endosymbiont *Wigglesworthia*, on CHC profiles and mate choice behavior. These findings highlight the importance of considering host-microbiota interactions when studying chemical communication and mate choice in insects.

**Electronic supplementary material:**

The online version of this article (10.1186/s12866-018-1292-7) contains supplementary material, which is available to authorized users.

## Background

Cuticular hydrocarbons (CHCs) are ubiquitous and both structurally and functionally diverse in insects [1]. Although the primary function of CHCs is the protection of the insect from water loss, they have secondarily adopted a multitude of functions in intra- and interspecific communication in a solitary as well as social context [1–5]. In particular, CHCs play an important role in mate attraction, species and sex recognition, courtship, and mate choice in many insect species [1, 6, 7].

Most insects are associated with obligate and/or facultative microbial symbionts that can affect physiology, ecology, and evolution of their hosts in a multitude of ways [8–10], including direct or indirect effects on chemical communication and mate choice [11]. Notably, experiments in locusts revealed a direct contribution of microbial gut symbionts to the production of the host’s cohesion pheromone [12, 13], and studies in fruit flies suggested that members of the microbiota can alter the CHC profile of the host and thereby affect mate choice decisions under certain circumstances [14–16]. Such pheromonal changes may constitute the first steps towards premating isolation and hence initiate speciation processes [11, 17].

Tsetse flies (*Glossina* spp., Diptera, Glossinidae) are associated with a taxonomically diverse microbial community. These microbes include environmentally acquired gut-associated microbes [18–20] as well as two bacterial symbionts (obligate mutualistic *Wigglesworthia glossinidia* and commensal *Sodalis glossinidius*) that are transmitted from pregnant females to their intrauterine larval offspring via maternal milk gland secretions [21, 22]. Some tsetse flies also house the reproductive symbiont *Wolbachia* [maternally transmitted through the germ line; 22] as well as viral and protozoan pathogens [23]. *Wigglesworthia* is an intracellular mutualist that serves important functions in tsetse, including supplementation of B-complex vitamins absent from vertebrate blood [24], and actuation of the development of tsetse’s immune system [25, 26]. While *Sodalis* is consistently present in flies, its function is not yet well established. *Wolbachia* is less prevalent, but is known to affect host reproduction across a wide range of insect hosts, including tsetse flies, where it causes cytoplasmic incompatibility [27–29].

While the effects of the microbial symbionts on tsetse fly metabolism and reproduction have been studied in detail, their possible impact on chemical communication and mate choice remains unknown. The CHCs of tsetse flies are characterized by a sex-specific blend of mono-, di-, and tri-methyl alkanes [30, 31]. Some of the long-chain methyl-branched CHCs have been implicated in eliciting sexual behavior of males upon contact with the females [32–36]. In *G. m. morsitans*, male contact with female-produced 15,19,23-trimethyl-heptatriacontane is necessary and sufficient to trigger male sexual behavior, provided that the compound is presented on a fly-like visual stimulus [32]. However, it remains elusive whether male CHCs also play a role for female mate choice decisions in tsetse.

Here, we set out to investigate the impact of bacterial symbionts on CHC profiles of *Glossina morsitans morsitans* and their possible influence on sexual selection and mating success. We used gas chromatography coupled to mass spectrometry (GC-MS) to analyze CHC profiles [37] of tsetse flies after antibiotic- as well as irradiation-mediated perturbations of the host-symbiont equilibrium [27, 38]. In addition, we assessed the effect of antibiotics on mating success of male and female *G. m. morsitans*.

## Methods

### Sampling and treatments

*Glossina morsitans morsitans* for antibiotic treatments were reared on bovine blood (Hemostat laboratories, Dixon, CA) in the laboratory at Yale University at 24 °C and on a 14 h/10 h light/dark photoregime. Two fly treatment groups were established by feeding pregnant females a diet supplemented with either ampicillin (Amp; 50 μg/ml blood; Pais et al., 2008) or tetracycline (Tet; 25 μg/ml blood; Alam et al., 2011). Tet-treated females were also supplemented with yeast extract [1% *w*/*v*; 24] to partially restore reproductive sterility that occurs in the absence of obligate *Wigglesworthia* [38]. Amp treatment of pregnant tsetse flies eliminates only *Wigglesworthia* from milk secretions such that larval offspring undergo their entire developmental program in the absence of this obligate symbiont but in the presence of *Sodalis* and *Wolbachia* [38]. Tet treatment eliminates all bacteria from pregnant females so that larvae undergo their entire developmental program in the absence of all bacteria [27]. Offspring from Amp and Tet treated mothers, which were used to test the impact of symbiont titer knockdown on tsetse’s CHC profile, are hereafter designated *Gmm*^*Wgm*-^ and *Gmm*^Apo^ (Apo = aposymbiotic), respectively. *Gmm*^*Wgm*-^ and *Gmm*^Apo^ flies were reared either collectively (experiment 1 and 2) or individually (experiment 3) on antibiotic-free bovine blood and sampled for chemical analyses. For experiment 3, only control and *Gmm*^Apo^ individuals were generated because rearing tsetse individually is untenable on a large scale. For each treatment group, 10 unmated male and 10 virgin female flies were sampled at day 10 (experiments 1 + 3) or day 5 (experiment 2) after adult emergence.

*Glossina morsitans morsitans* flies used for irradiation treatments were originally from Zimbabwe and maintained since 1997 at the Insect Pest Control Laboratory (IPCL) of the Joint FAO/IAEA Division of Nuclear Techniques in Food and Agriculture, Seibersdorf, Austria. Tsetse flies were maintained at a temperature of 23 ± 1 °C, a relative humidity of 75–80% under on a 12 h/12 h light/dark photoregime. Experimental flies were fed on defibrinated bovine blood using an artificial (in vitro) membrane feeding system for 15–20 min three times per week [39]. Male flies were either irradiated as 22-day old pupae (early), at the very late pupal stage at which females had already emerged (29-days old (late)), or as 5-day old adults (adult). The irradiation treatment was performed using a Gammacell 220 60Co irradiator (Nordion Ltd., Ottawa, Canada) by exposing the samples for different time periods to receive an irradiation dose of 110, 50 or 20 Gy. Non irradiated flies were used as a control (0 Gy). Twenty two-day and 29-day old pupae were irradiated in a 9 cm diameter petri-dish while the 5-day old males were irradiated in individual small cages (4 cm diameter × 6 cm high) (one male/cage). Irradiated pupae were separated and reared individually in a pill sorter until emergence. After emergence each male was individually placed in a small cage and maintained until day 10 after emergence. Depending on the time and dosage, irradiation treatment has variable effects on *Sodalis* and *Wolbachia*, but not *Wigglesworthia* titers [40]. Specifically, in adult flies emerging from early irradiated (22-day old) pupae, *Sodalis* density was decreased at 24 h post emergence and recovered over time until day 14 post eclosure, while the *Wigglesworthia* titer did not differ between treatment and control groups, and *Wolbachia* density was increased at emergence but decreased again over time. In the males emerging from late irradiated (29-day old) pupae, both *Sodalis* and *Wolbachia* density was reduced during the first week after emergence and then recovered over time. In males irradiated as adults, *Sodalis* density decreased after irradiation while the *Wolbachia* density increased at 24 h post irradiation and then decreased again over time.

### Extraction of samples and GC-MS analysis

Individual flies were extracted in hexane. 2 μg of heneicosane was added as internal standard to allow for later quantification of hydrocarbons. Extracts were evaporated to about 20-30 μl of hexane under a constant stream of argon and transferred to a 150 μl GC-μ-vial (CZT, Kriftel, Germany) for Gaschrommatography-Massspectrometry analysis. An aliquot of 1 μl of each sample was injected into a Varian 450GC gas chromatograph coupled to a Varian 240MS mass spectrometer (Agilent Technologies, Böblingen, Germany) using a split/splitless injector at 250 °C with the purge valve opened after 60s. The GC was equipped with a DB5-MS capillary column (30 m × 0.25 mm diameter, film thickness: 0.25 μm, Agilent Technologies) and programmed from 150 to 300 °C at 15 °C/min with a 27 min. final isothermal hold. Helium was used as carrier gas, with a constant flow rate of 1 ml/min. Mass spectra were recorded using electron impact ionization (EI-MS). Data acquisition and quantifications were achieved with MS Workstation Version 6.9.3 Software (Agilent Technologies). The peaks were identified by their mass spectra in comparison to previously published analyses of *G. m. morsitans* cuticular hydrocarbon profiles [30]. Peak areas were automatically integrated using the MS Workstation Software. Finally, the success of this integration was controlled manually for every peak. Some substances had to be combined for the analysis, as the peaks were not always clearly separated in the chromatograms.

### Mate choice assays

Individual control males or females were given a simultaneous choice between one control and one *Gmm*^Apo^ mate, respectively. All flies were 5 days old adults. To later distinguish the individuals, the last tarsal segment was cut from either the right or left mid leg. The control and *Gmm*^Apo^ mate were set up in the clean round colony cage with 20 cm of diameter and height of 5 cm, 1 day post feeding and 6 h before the actual experiment. An individual control male/female was inserted into the middle of the cage while the potential mates were held on the opposite side of cage by shading them with a black blanket. After the removal of the blanket, the control fly was given the ability to come into contact with both potential mates before choosing a mating partner. Matings were scored visually by observing the cage for 3 h or until the end of a successful mating, which lasts in *G. m. morsitans* 2 h [41]. For male choice, 30 replicates were performed, while 17 replicates were done for female choice assays due to the availability of flies.

### Statistical analysis

Since CHC profiles of tsetse flies are sex-specific, the profiles of males and females were analyzed separately. To compare absolute amounts of hydrocarbons across treatment groups, the total amount of all compounds (combined) was calculated from the combined peak areas by comparison to the peak area of the internal standard (=2 μg). For the known contact sex pheromone of female *G. m. morsitans*, 15,19,23-trimethyl-heptatriacontane [32], absolute and relative amounts were calculated for each individual, based on the internal standard and the total peak area of all hydrocarbons, respectively. The resulting values were compared among antibiotic treatment groups by ANOVA with Tukey post-hoc comparisons. Irradiation treatment groups were analyzed in a two-factorial ANOVA to test for effects of the dosage and age/developmental stage at which the flies were subjected to irradiation.

For all other analyses, relative amounts were calculated from the peak areas and then log-ratio-transformed according to Aitchison [42]. In order to test for differences in chemical profiles across groups, principal component analyses (PCAs) were performed to reduce the number of variables, and the resulting PCs (with Eigenvalues > 0.9) were used for discriminant analyses (DAs) to test for among-group differences. Chi-squared tests were performed for the mate choice assays. All statistical analyses were done with SPSS 17.0.

## Results

### CHC composition in G. m. Morsitans

As described earlier [30], CHC profiles of *G. m. morsitans* were dominated by mono-, di-, and tri-methyl alkanes, and there were distinct sex-specific differences, with females generally showing more compounds with longer carbon backbones (Tables 1, 2 and 3). The main components of female CHC profiles were 2-methyl-triacontane, 15,19- and 17,21-dimethyl-heptatriacontane, and 15,19,23-trimethyl-heptatriacontane, which together accounted for about 70% of the complete CHCs in control flies (Table 1). In males, 2-methyl-triacontane and 11,15-dimethyl-tritriacontane dominated, amounting to about 40% of the total CHC profile in control flies from Vienna and 70% in control flies from Yale (Tables 2, 3). In addition to these differences in the dominant compounds, males reared in Vienna showed slightly more of the longer carbon backbone compounds then males reared at Yale (Tables 2, 3).

**Table 1:** CHC profiles of 5- and 10-day old antibiotic-treated (*Gmm*^Wgm-^ = ampicillin; *Gmm*^Apo^ = tetracycline) and control female *G. m. morsitans* after mass- or individual rearing. Compounds are sorted by class (mono-, di-, and trimethyl-alkanes). Given are average relative amounts of CHCs (in percent) +/− standard deviation, as well as the total absolute amount of CHCs as determined by comparison with an internal standard. Me = methyl

Compound	5-day old, mass-rearing			10-day old, mass-rearing			10-day old, individual rearing	
	Control	*Gmm* ^Wgm-^	*Gmm* ^Apo^	Control	*Gmm* ^Wgm-^	*Gmm* ^Apo^	Control	*Gmm* ^Apo^
2Me-C28	0.41 ± 0.13	0.58 ± 0.19	0.83 ± 0.62	0.28 ± 0.11	0.80 ± 0.70	0.75 ± 0.40	0.46 ± 0.14	1.16 ± 0.32
2Me-C29	0.77 ± 0.23	1.81 ± 0.60	1.32 ± 0.76	1.26 ± 0.42	1.78 ± 0.70	1.89 ± 0.45	1.88 ± 0.70	3.56 ± 0.61
2Me-C30	13.16 ± 2.71	21.57 ± 6.70	13.89 ± 5.51	20.72 ± 4.04	20.75 ± 4.21	26.15 ± 9.91	31.02 ± 6.64	37.94 ± 3.87
2Me-C31	0.47 ± 0.20	0.80 ± 0.32	0.57 ± 0.29	0.63 ± 0.16	0.80 ± 0.78	0.97 ± 0.48	1.20 ± 0.38	2.19 ± 1.00
2Me-C32	0.89 ± 0.25	1.55 ± 0.50	1.13 ± 0.65	0.89 ± 0.26	0.90 ± 0.30	1.12 ± 0.36	1.47 ± 0.32	2.09 ± 0.70
2Me-C34	0.08 ± 0.05	0.14 ± 0.12	0.10 ± 0.07	0.06 ± 0.04	0.12 ± 0.07	0.11 ± 0.07	0.05 ± 0.02	0.12 ± 0.08
11,15-diMe-C33	0.46 ± 0.14	1.04 ± 0.32	0.96 ± 0.53	0.43 ± 0.13	0.46 ± 0.33	0.99 ± 1.23	0.18 ± 0.04	0.82 ± 0.70
diMe-C34	0.36 ± 0.09	0.44 ± 0.09	0.45 ± 0.14	0.20 ± 0.04	0.22 ± 0.10	0.24 ± 0.21	0.32 ± 0.07	0.34 ± 0.16
15,19-diMe-C35	9.13 ± 1.31	8.51 ± 1.15	8.06 ± 1.33	5.54 ± 0.56	5.42 ± 0.08	5.62 ± 2.51	7.76 ± 0.98	5.84 ± 1.71
15,19 + 16,20-diMe-C36	6.85 ± 0.54	6.35 ± 0.43	7.03 ± 0.77	4.35 ± 0.54	3.74 ± 0.45	4.43 ± 1.22	5.66 ± 0.66	4.32 ± 0.71
15,19 + 17,21-diMe-C37	21.80 ± 3.82	17.61 ± 2.71	17.60 ± 2.35	13.72 ± 1.89	13.30 ± 3.84	15.11 ± 3.36	13.67 ± 2.63	10.19 ± 2.21
diMe-C38	1.57 ± 0.19	1.47 ± 0.24	1.83 ± 0.58	0.90 ± 0.21	0.66 ± 0.30	1.06 ± 0.23	0.74 ± 0.22	0.64 ± 0.12
13,17,21-triMe-C35	0.64 ± 0.15	0.51 ± 0.08	0.69 ± 0.10	0.57 ± 0.13	1.19 ± 1.27	0.42 ± 0.14	0.85 ± 0.19	0.96 ± 0.13
triMe-C36	1.86 ± 0.44	1.65 ± 0.29	2.17 ± 0.33	1.81 ± 0.36	1.85 ± 0.63	1.39 ± 0.35	2.16 ± 0.53	1.94 ± 0.28
15,19,23-triMe-C37	27.35 ± 3.18	23.05 ± 2.69	27.85 ± 3.55	36.58 ± 2.52	37.67 ± 6.04	28.88 ± 7.64	25.10 ± 3.29	21.91 ± 2.63
15,19,23-triMe-C38	8.40 ± 1.42	7.90 ± 1.51	9.93 ± 2.06	7.88 ± 1.83	6.76 ± 1.86	7.01 ± 0.55	6.52 ± 1.82	5.14 ± 0.98
15,19,23-triMe-C39	5.41 ± 1.16	4.46 ± 1.32	5.24 ± 1.58	3.78 ± 1.00	2.89 ± 0.92	3.43 ± 0.67	0.24 ± 0.15	0.19 ± 0.06
unknown	0.37 ± 0.15	0.53 ± 0.22	0.36 ± 0.15	0.41 ± 0.19	0.70 ± 0.68	0.45 ± 0.27	0.70 ± 0.30	0.63 ± 0.20
Total CHC amount (μg)	35.04 ± 9.36	29.70 ± 26.66	43.32 ± 18.73	33.43 ± 29.28	24.17 ± 20.06	18.48 ± 14.72	26.98 ± 10.48	28.22 ± 6.44

**Table 2:** CHC profiles of 5- and 10-day old antibiotic-treated (*Gmm*^Wgm-^ = ampicillin; *Gmm*^Apo^ = tetracycline) and control male *G. m. morsitans* after mass- or individual rearing. Compounds are sorted by class (mono-, di-, and trimethyl-alkanes). Given are average relative amounts of CHCs (in percent) +/− standard deviation, as well as the total absolute amount of CHCs as determined by comparison with an internal standard. Me = methyl

Compound	5-day old, mass-rearing			10-day old, mass-rearing			10-day old, individual rearing	
	Control	*Gmm* ^Wgm-^	*Gmm* ^Apo^	Control	*Gmm* ^Wgm-^	*Gmm* ^Apo^	Control	*Gmm* ^Apo^
2Me-C28	0.42 ± 0.18	1.36 ± 0.91	1.20 ± 0.37	0.45 ± 0.18	0.84 ± 0.42	2.15 ± 0.89	0.58 ± 0.10	1.29 ± 0.49
2Me-C29	4.36 ± 1.03	6.21 ± 2.17	5.74 ± 1.00	8.83 ± 1.77	8.10 ± 3.03	7.59 ± 0.52	8.80 ± 1.11	9.24 ± 1.69
2Me-C30	19.52 ± 1.67	21.89 ± 3.40	21.81 ± 1.70	20.42 ± 2.82	22.96 ± 2.01	29.22 ± 4.91	22.12 ± 2.07	29.23 ± 4.85
2Me-C31 + 9,13-diMe-C31	4.39 ± 075.	5.59 ± 1.70	4.62 ± 0.52	3.79 ± 0.24	5.11 ± 0.87	5.03 ± 0.87	6.54 ± 0.40	7.46 ± 1.70
2Me-C32 + diMe-C32	4.47 ± 0.37	5.79 ± 0.82	5.48 ± 0.43	3.32 ± 0.35	3.78 ± 0.48	4.40 ± 0.25	5.57 ± 0.67	5.73 ± 1.32
11,15-diMe-C33	49.51 ± 2.60	37.92 ± 7.16	39.70 ± 2.55	51.62 ± 3.77	46.12 ± 4.19	36.18 ± 3.99	47.04 ± 2.04	36.00 ± 9.06
12,16-diMe-C34	5.59 ± 0.47	5.85 ± 1.02	5.20 ± 0.31	3.30 ± 0.47	2.96 ± 0.51	3.77 ± 0.51	4.24 ± 0.65	3.53 ± 0.60
15,19-diMe-C35 + 8-Me-C35:1	9.28 ± 2.61	11.70 ± 2.03	12.64 ± 2.40	5.36 ± 0.94	5.09 ± 0.93	8.17 ± 1.53	1.96 ± 0.45	2.07 ± 0.84
7,11,15-triMe-C33	1.06 ± 0.18	1.45 ± 0.37	1.48 ± 0.21	0.91 ± 0.17	2.00 ± 0.91	1.27 ± 0.22	1.10 ± 0.19	1.78 ± 0.86
unknown1	0.11 ± 0.08	0.18 ± 0.09	0.13 ± 0.08	0.75 ± 0.38	0.10 ± 0.09	0.23 ± 0.08	0.29 ± 0.12	0.32 ± 0.12
unknown2	0.39 ± 0.10	0.61 ± 0.15	0.62 ± 0.07	0.37 ± 0.07	1.17 ± 0.68	0.69 ± 0.09	0.62 ± 0.15	1.56 ± 1.11
unknown3	0.28 ± 0.05	0.38 ± 0.13	0.38 ± 0.04	0.20 ± 0.04	0.52 ± 0.22	0.30 ± 0.09	0.37 ± 0.10	0.65 ± 0.40
unknown4	0.62 ± 0.20	1.07 ± 0.32	0.99 ± 0.19	0.68 ± 0.05	1.26 ± 0.46	0.99 ± 0.11	0.77 ± 0.12	1.14 ± 0.49
Total CHC amount (μg)	17.17 ± 7.91	20.95 ± 7.10	15.89 ± 4.53	25.04 ± 12.89	19.62 ± 10.43	4.30 ± 1.82	38.29 ± 8.94	36.25 ± 19.02

**Table 3:** CHC profiles of male *Glossina m. morsitans* treated with different gamma-irradiation doses at three time points (early and late pupae, and young adults, respectively). Compounds are sorted by class (mono-, di-, and trimethyl-alkanes). Given are average relative amounts of CHCs (in percent) +/− standard deviation, as well as the total absolute amount of CHCs as determined by comparison with an internal standard. Me = methyl

Compound	Irradiated as early pupae				Irradiated as late pupae				Irradiated as young adults			
	0Gy	20Gy	50Gy	110Gy	0Gy	20Gy	50Gy	110Gy	0Gy	20Gy	50Gy	110Gy
2Me-C28	0.54 ± 0.44	0.37 ± 0.19	0.62 ± 0.46	0.79 ± 0.88	0.77 ± 0.98	0.96 ± 1.11	0.78 ± 0.6	0.68 ± 1.1	1.09 ± 1.59	0.62 ± 0.6	0.91 ± 0.97	0.81 ± 0.82
2Me-C29	8.74 ± 2.18	9.28 ± 1.24	9.74 ± 2.07	9.1 ± 1.79	8.4 ± 3.57	8.07 ± 2.02	8.59 ± 2.92	7.18 ± 2.19	8.48 ± 2.28	9.46 ± 1.63	9.1 ± 1.09	9.84 ± 2.51
2Me-C30	20.64 ± 4.4	20.74 ± 2.49	23.11 ± 3.66	21.95 ± 3.29	24.62 ± 5.23	22.38 ± 5.71	21.76 ± 5.94	19.6 ± 7.91	23.88 ± 8.78	22.61 ± 3.14	21.79 ± 4.14	23.15 ± 4.83
2Me-C31 + 9,13-diMe-C31	6.08 ± 0.91	6.14 ± 0.97	6.1 ± 0.81	5.8 ± 0.94	5.84 ± 0.85	5.55 ± 0.65	5.96 ± 0.89	5.62 ± 1.4	6.19 ± 1.24	6.26 ± 0.67	6.34 ± 0.62	6.08 ± 0.44
2Me-C32 + diMe-C32	4.52 ± 0.56	4.67 ± 0.65	3.96 ± 0.52	4.46 ± 0.68	4.41 ± 0.78	4.49 ± 0.65	4.82 ± 1.3	4.7 ± 0.82	4.53 ± 0.89	4.43 ± 0.58	4.59 ± 0.71	4.09 ± 0.51
11,15-diMe-C33	47.6 ± 4.02	47.49 ± 2.75	46.07 ± 4.83	46.56 ± 3.72	45.02 ± 4.17	47.63 ± 4.79	44.95 ± 5.86	47.46 ± 12.05	44.48 ± 7.63	45.47 ± 3.97	45.52 ± 2.74	43.91 ± 6.32
12,16-diMe-C34	3.21 ± 0.47	3.15 ± 0.38	2.88 ± 0.27	3.09 ± 0.73	3.01 ± 0.48	3.15 ± 0.5	3.46 ± 0.82	3.62 ± 1.01	3.06 ± 0.65	3.08 ± 0.3	3.21 ± 0.61	3.06 ± 0.62
15,19-diMe-C35 + 8-Me-C35:1	4.73 ± 0.98	4.22 ± 0.72	4.42 ± 0.75	4.88 ± 0.69	4.32 ± 1.03	4.08 ± 0.85	5.29 ± 0.86	5.41 ± 1.3	4.56 ± 1.47	3.98 ± 0.45	4.24 ± 1.13	4.6 ± 0.77
15,19 + 16,20-diMe-C36	0.17 ± 0.1	0.13 ± 0.07	0.14 ± 0.03	0.17 ± 0.05	0.17 ± 0.07	0.16 ± 0.04	0.2 ± 0.04	0.39 ± 0.7	0.15 ± 0.05	0.11 ± 0.03	0.13 ± 0.03	0.16 ± 0.08
15,19 + 17,21-diMe-C37	0.12 ± 0.09	0.06 ± 0.03	0.12 ± 0.06	0.12 ± 0.05	0.13 ± 0.05	0.12 ± 0.05	0.17 ± 0.08	0.96 ± 2.69	0.12 ± 0.09	0.09 ± 0.04	0.09 ± 0.03	0.11 ± 0.06
7,11,15-triMe-C33	0.92 ± 0.18	0.93 ± 0.12	0.77 ± 0.16	0.88 ± 0.2	0.91 ± 0.35	0.93 ± 0.15	1.12 ± 0.32	0.95 ± 0.3	0.86 ± 0.21	0.95 ± 0.11	1.06 ± 0.21	1.06 ± 0.22
15,19,23-triMe-C37	0.06 ± 0.06	0.03 ± 0.02	0.04 ± 0.02	0.04 ± 0.03	0.05 ± 0.03	0.05 ± 0.03	0.06 ± 0.03	0.84 ± 2.51	0.05 ± 0.06	0.03 ± 0.01	0.03 ± 0.02	0.07 ± 0.06
unknown1	1.21 ± 0.53	1.34 ± 0.42	0.77 ± 0.49	0.76 ± 0.41	0.84 ± 0.55	0.91 ± 0.55	0.97 ± 0.6	0.92 ± 0.33	1.06 ± 0.92	1.2 ± 0.46	1.1 ± 0.51	1.27 ± 0.7
unknown2	0.47 ± 0.18	0.47 ± 0.12	0.42 ± 0.12	0.46 ± 0.15	0.54 ± 0.29	0.53 ± 0.15	0.61 ± 0.19	0.46 ± 0.12	0.46 ± 0.09	0.52 ± 0.11	0.65 ± 0.19	0.56 ± 0.14
unknown3	0.27 ± 0.07	0.26 ± 0.08	0.22 ± 0.05	0.24 ± 0.06	0.28 ± 0.12	0.29 ± 0.04	0.33 ± 0.13	0.29 ± 0.09	0.27 ± 0.07	0.29 ± 0.05	0.3 ± 0.09	0.27 ± 0.06
unknown4	0.73 ± 0.15	0.72 ± 0.12	0.62 ± 0.12	0.7 ± 0.14	0.68 ± 0.19	0.7 ± 0.09	0.94 ± 0.29	0.94 ± 0.27	0.77 ± 0.21	0.9 ± 0.13	0.94 ± 0.19	0.95 ± 0.21
Total CHC amount (μg)	33.48 ± 11.08	34.83 ± 11.81	32.02 ± 9.02	35.22 ± 11.92	35.26 ± 12.03	26.06 ± 10.17	37.03 ± 21.52	31.60 ± 12.73	24.17 ± 10.09	31.01 ± 8.98	27.63 ± 8.21	25.20 ± 12.97

### Influence of antibiotic treatment on CHC profiles in mass-reared female flies

**Fig. 1:**
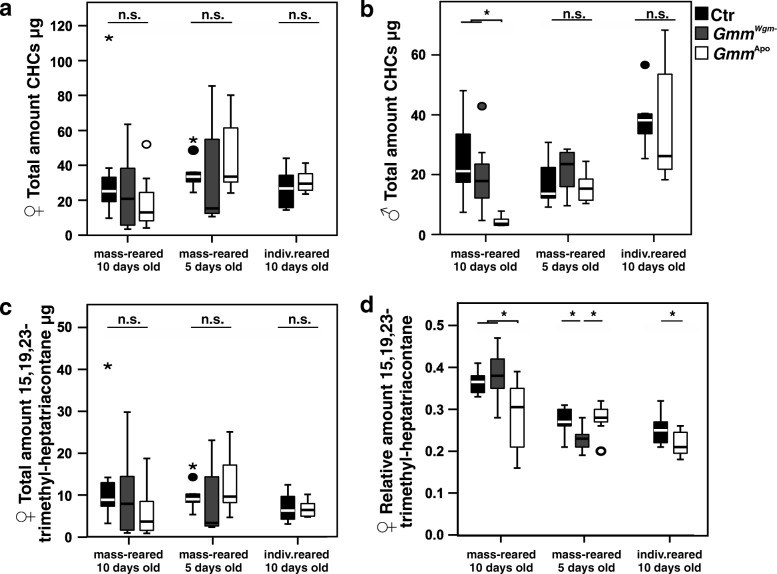
Effect of antibiotic treatment on the total amount of hydrocarbons in female (**a**) and male (**b**) *Glossina m. morsitans*, as well as on the absolute (**c**) and relative (**d**) amount of the females’ contact sex pheromone 15,19,23-trimethyl-heptatriacontane. Ctr = Control (without antibiotics), *Gmm*^Wgm-^ = ampicillin-treated, *Gmm*^Apo^ = tetracycline-treated. Lines represent medians, boxes comprise interquartile ranges, and whiskers denote minimum and maximum values, except for outliers that lie further away from a quartile than 1.5 times (circles) or 3 times (asterisks) the interquartile range. An asterisk above lines connecting single treatments indicates a significant difference at *p* < 0.05

Antibiotic treatment had no effect on the total amount of CHCs in 10-day-old females (Fig. 1a; ANOVA, F_2,27_ = 1.154, *p* = 0.330). *Gmm*^Apo^ females showed a non-significant tendency towards lower absolute amounts of 15,19,23-trimethyl-heptatriacontane (Fig. 1c; ANOVA, F_2,27_ = 1.267, *p* = 0.298). A comparison of the relative amounts of 15,19,23-trimethyl-heptatriacontane revealed significantly lower proportions of sex pheromone in *Gmm*^Apo^ females as compared to control and *Gmm*^*Wgm*-^ flies (Fig. 1d; ANOVA, F_2,27_ = 6.291, *p* = 0.006).

**Fig. 2:**
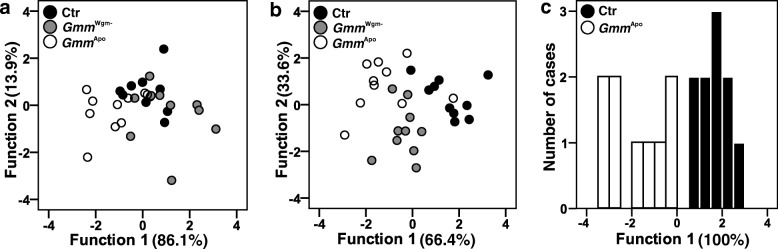
Effect of antibiotic treatment on CHC profiles of female tsetse flies (*G. m. morsitans*). Discriminant analyses based on log-ratio transformed relative amounts of (**a**) CHCs of mass-reared, 10 day old females, (**b**) mass-reared 5 day old females, and (**c**) individually reared, 10 day old females. Ctr = Control (without antibiotics), *Gmm*^Wgm-^ = ampicillin-treated, *Gmm*^Apo^ = tetracycline-treated

Based on the 19 quantified peaks, four principal components were extracted, capturing 83.9% of the total variance. A discriminant analysis (DA) based on the four PCs including all three treatment groups yielded a significant difference in CHC profiles across groups (Fig. 2a; Wilks’ Lambda = 0.354, X^2^ = 26.5, df = 8, *p* = 0.001). Based on the two discriminant functions, 60% of the cases were correctly classified (30% would be expected by chance). Subsequent DAs of pairwise combinations of the three groups revealed no significant difference between control and *Gmm*^*Wgm*-^flies (Wilks’ Lambda = 0.595, X^2^ = 8.30, df = 4, *p* = 0.081), but significant differences between control and *Gmm*^Apo^ flies (Wilks’ Lambda = 0.498, X^2^ = 11.1, df = 4, *p* = 0.025) and between *Gmm*^*Wgm*-^ and *Gmm*^Apo^ flies (Wilks’ Lambda = 0.402, X^2^ = 14.6, df = 4, *p* = 0.006), respectively.

In 5-day-old females, there was also no difference in total amount of CHCs across groups (Fig. 1a; ANOVA, F_2,27_ = 1.234, *p* = 0.307). *Gmm*^*Wgm*-^ females showed a non-significant tendency towards lower absolute amounts of 15,19,23-trimethyl-heptatriacontane (Fig. 1c; ANOVA, F_2,27_ = 1.785, *p* = 0.187). A comparison of the relative amounts of 15,19,23-trimethyl-heptatriacontane revealed significantly lower proportions of sex pheromone in *Gmm*^*Wgm*-^ females as compared to control and *Gmm*^Apo^ flies (Fig. 1d; ANOVA, F_2,27_ = 6.981, *p* = 0.004; Tukey HSD *p* = 0.014 for control-*Gmm*^*Wgm*-^ and *p* = 0.001 for *Gmm*^*Wgm*-^-*Gmm*^Apo^).

Based on the 18 quantified peaks, four principal components were extracted, capturing 85.0% of the total variance. A discriminant analysis (DA) based on the four PCs including all three treatment groups yielded a significant difference in CHC profiles across groups (Fig. 2b; Wilks’ Lambda = 0.224, X^2^ = 38.1, df = 8, *p* < 0.001). Based on the two discriminant functions, 83.3% of the cases were correctly classified (30% would be expected by chance). Subsequent DAs of pairwise combinations of the three groups revealed a significant difference between control and *Gmm*^*Wgm*-^ flies (Wilks’ Lambda = 0.232, X^2^ = 23.37, df = 4, *p* < 0.001), between control and *Gmm*^Apo^ flies (Wilks’ Lambda = 0.367, X^2^ = 16.0, df = 4, *p* = 0.003) and also between *Gmm*^*Wgm*-^- and *Gmm*^Apo^ flies (Wilks’ Lambda = 0.405, X^2^ = 14.5, df = 4, *p* = 0.006), respectively.

### Influence of antibiotic treatment on CHC profiles in mass-reared male flies

**Fig. 3:**
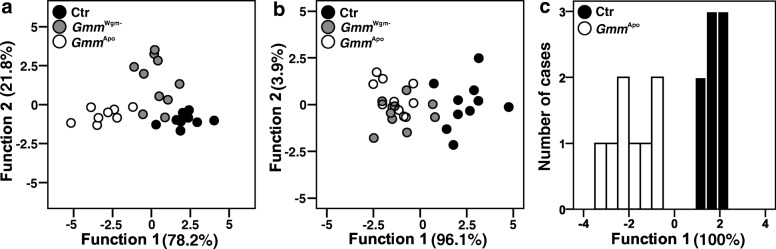
Effect of antibiotic treatment on CHC profiles of male tsetse flies (*G. m. morsitans*). Discriminant analyses based on log-ratio transformed relative amounts of (**a**) CHCs of mass-reared, 10 day old males, (**b**) mass-reared 5 day old males, and (**c**) individually reared, 10 day old males. Ctr = Control (without antibiotics), *Gmm*^Wgm-^ = ampicillin-treated, *Gmm*^Apo^ = tetracycline-treated

In 10-day-old male flies, control and *Gmm*^*Wgm*-^ individuals showed on average 5–6 times higher total amounts of CHCs than did *Gmm*^Apo^ flies (Fig. 1b; ANOVA, F_2,25_ = 10.03, *p* = 0.001). Post-hoc comparisons (Tukey HSD) revealed these differences to be significant (*p* = 0.001 and *p* = 0.009 for control-*Gmm*^Apo^ and *Gmm*^*Wgm*-^-*Gmm*^Apo^, respectively), while there was no difference between control and *Gmm*^*Wgm*-^ flies (*p* = 0.457). Based on the 13 quantified peaks, four principal components were extracted, capturing 84.3% of the total variance. A discriminant analysis (DA) based on the four PCs including all three treatment groups yielded a significant difference in CHC profiles across groups (Fig. 3a; Wilks’ Lambda = 0.046, X^2^ = 72.5, df = 8, *p* < 0.001). Based on the two discriminant functions, 96.4% of the cases were correctly classified (30% would be expected by chance). Subsequent DAs of all pairwise combinations of the three groups revealed significant differences between all groups: control vs. *Gmm*^*Wgm*-^: Wilks’ Lambda = 0.233, X^2^ = 23.3, df = 4, *p* < 0.001; control vs. *Gmm*^Apo^: Wilks’ Lambda = 0.076, X^2^ = 36.0, df = 4, *p* < 0.001; *Gmm*^*Wgm*-^- vs. *Gmm*^Apo^: Wilks’ Lambda = 0.177, X^2^ = 24.2, df = 4, *p* < 0.001.

Antibiotic treatment had no effect on the total amount of CHCs in 5-day-old males (Fig. 1b; ANOVA, F_2,27_ = 1.565, *p* = 0.227). Based on the 13 quantified peaks, five principal components were extracted, capturing 88.8% of the total variance. A discriminant analysis (DA) based on the four PCs including all three treatment groups yielded a significant difference in CHC profiles across groups (Fig. 3b; Wilks’ Lambda = 0.207, X^2^ = 39.3, df = 10, *p* < 0.001). Based on the two discriminant functions, 70.0% of the cases were correctly classified (30% would be expected by chance). Subsequent DAs of all pairwise combinations of the three groups revealed significant differences between control vs. *Gmm*^*Wgm*-^ males (Wilks’ Lambda = 0.253, X^2^ = 21.3, df = 5, *p* < 0.001); control vs. *Gmm*^Apo^ males (Wilks’ Lambda = 0.146, X^2^ = 29.8, df = 5, *p* < 0.001), but not between *Gmm*^*Wgm*-^ and *Gmm*^Apo^ males (Wilks’ Lambda = 0.727, X^2^ = 4.9, df = 5, *p* < 0.424).

### Influence of antibiotic treatment in individually reared flies

In individually reared 10-day-old females, there was no difference in the total amount of CHCs between control and *Gmm*^Apo^ flies (Fig. 1a; t-test, *T* = − 0.888, df = 16, *p* = 0.388), nor in the absolute amount of female sex pheromone (Fig. 1c; t-test, *T* = 0.170, df = 16, *p* = 0.868). A comparison of the relative amounts of 15,19,23-trimethyl-heptatriacontane revealed significantly lower proportions of sex pheromone in *Gmm*^Apo^ females as compared to control flies (Fig. 1d; t-test, *T* = 2.080, df = 17, *p* = 0.044). Based on the 18 quantified peaks, four principal components were extracted, capturing 87.2% of the total variance. A discriminant analysis (DA) based on the four PCs including yielded a significant difference in CHC profiles between control and *Gmm*^Apo^ females (Fig. 2c; Wilks’ Lambda = 0.233, X^2^ = 21.8, df = 4, *p* < 0.001). Based on the first discriminant functions, 94.7% of the cases were correctly classified (50% would be expected by chance).

As in females, individually reared 10-day-old males showed no difference in the total amount of CHCs between control and *Gmm*^Apo^ flies (Fig. 1b; t-test for non-equal variances, *T* = 0.287, df = 11.653, *p* = 0.779). Based on the 13 quantified peaks, four principal components were extracted, capturing 87.2% of the total variance. A discriminant analysis (DA) based on the four PCs yielded a significant difference in CHC profiles across groups (Fig. 3c; Wilks’ Lambda = 0.246, X^2^ = 18.2, df = 4, *p* = 0.0011). Based on one discriminant function, 94.1% of the cases were correctly classified (50% would be expected by chance).

### Comparison of CHC profiles between antibiotic experiments

A comparison of chemical profiles of *Gmm*^Apo^ and control flies between the three experiments revealed significant differences between treatments and experiments for both females (Additional file 1a; Wilks’ Lambda = 0.027, X^2^ = 192.2, df = 20, *p* < 0.001) and males (Additional file 1b; Wilks’ Lambda = 0.005, X^2^ = 250.6, df = 20, *p* < 0.001). In particular, fly profiles were very well separated into the three experiments in the discriminant analysis.

### Influence of gamma-irradiaton on male flies

**Fig. 4:**
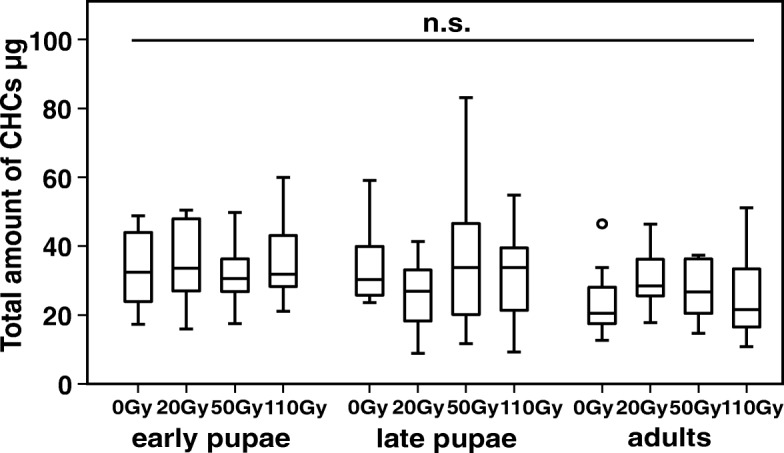
Effect of gamma-irradiation on the total amount of CHCs in male *Glossina m. morsitans* flies. 0Gy = control (without irradiation). Male flies were irradiated at one of three different time points (early pupae, age 22 days; late pupae, 29 days; or young adults, 5 days). Lines represent medians, boxes comprise interquartile ranges, and whiskers denote minimum and maximum values, except for outliers that lie further away from a quartile than 1.5 times (circles) or 3 times (asterisks) the interquartile range

In a full factorial model, no overall differences in total CHC amounts could be detected between treatment groups (ANOVA F_11,108_ = 1.292, *p* = 0.239; time points: F_2,108_ = 3.577, *p* = 0.031; irradiation dose: F_3,108_ = 0.114, *p* = 0.952; interaction: F_6,108_ = 1.119, *p* = 0.356; Fig. 4).

**Fig. 5:**
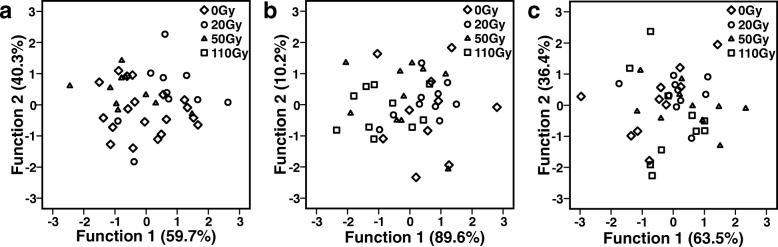
Effect of gamma-irradiation dose on CHC profiles of male tsetse flies (*G. m. morsitans*). Discriminant analyses based on log-ratio transformed relative amounts of individually reared 10 day old males that were treated with different irradiation doses (0Gy, 20Gy, 50Gy, 110Gy) (**a**) during early pupal development (at day 22), (**b**) during late pupal development (after females emerged) and (**c**) to adult males (day 5 post-eclosion)

Based on the 16 quantified peaks and the 12 treatment groups, three principal components were extracted, capturing 80.0% of the total variance. A discriminant analysis (DA) based on the three PCs including all twelve treatment groups yielded a significant difference in CHC profiles across groups (Wilks’ Lambda = 0.592, X^2^ = 58.5, df = 33, *p* = 0.004; Additional file 2), but only 28.3% of the cases were classified correctly based on both discriminant functions. When treatment time point was used as a grouping variable, the groups also differed significantly (Wilks’ Lambda = 0.781, X^2^ = 28.7, df = 6, *p* < 0.001; Additional file 3), with 60% of the cases being classified correctly. Subsequent DAs of irradiation treatments at single time points revealed no significant difference between irradiation treatments for any of the three time points (including the 0Gy control; early: Wilks’ Lambda = 0.677, X^2^ = 13.9, df = 9, *p* = 0.127; late: Wilks’ Lambda = 0.693, X^2^ = 13.0, df = 6, *p* = 0.126; adult: Wilks’ Lambda = 0.799, X^2^ = 8.0, df = 6, *p* = 0.539; Fig. 5a-c).

### Mate choice assays with mass-reared flies

**Fig. 6:**
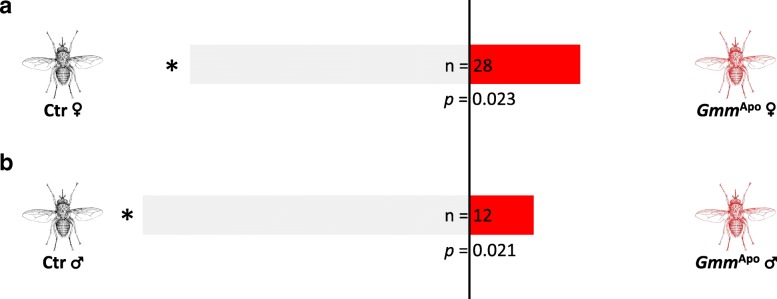
Effect of tetracycline treatment on mating success of 5 day old (**a**) male and (**b**) female tsetse flies (*G. m. morsitans*). An untreated individual of the opposite sex was given a simultaneous choice between a *Gmm*^Apo^ and an untreated individual. Ctr = Control (without antibiotics), *Gmm*^Apo^ = tetracycline-treated

Out of the 30 male mate choice assays, the males chose females with their native microbiota in 20 cases and *Gmm*^Apo^ females in 8 cases (Fig. 6a). Two males remained unmated. Excluding the unmated males, this distribution differs significantly from random mating (df = 1, Chi^2^ = 5.14, *p* = 0.02). Of the 17 females, 10 mated with males with their native microbiota, two with *Gmm*^Apo^ males, and five remained unmated (Fig. 6b). Excluding the unmated females, this distribution differed significantly from random mating (df = 1, Chi^2^ = 5.33, *p* = 0.02).

## Discussion

We assessed the impact of antibiotic treatment on the CHC profiles and mating success of male and female tsetse flies. Neither the absolute amount of all CHCs in females, nor the absolute amount of the female sex pheromone 15,19,23-trimethyl-heptatriacontane was effected by Amp or Tet treatment under any rearing condition. However, the relative amount of the sex pheromone was significantly reduced after Tet treatment. In males, the total amount of CHCs was significantly reduced in mass-reared *Gmm*^Apo^ males, but not in *Gmm*^*Wgm*-^ and individually reared *Gmm*^Apo^ males. The CHC profiles of both females and males differed significantly between treatments under all rearing conditions except for mass reared *Gmm*^*Wgm*-^ vs. *Gmm*^Apo^ 5-day old males. Further, gamma-irradiation of male pupae or young adults did not affect the CHC profile of 10-day old males, even though a previous study has shown that the treatment with 110Gy causes significant, yet variable effects on the three symbiont titers, based on irradiation time [40]. Finally, both male and female flies with their native microbiota discriminated against *Gmm*^Apo^ flies in mate choice assays.

The bacterial symbionts harbored by tsetse flies exhibit differential sensitivity to antibiotics and irradiation. Only *Wigglesworthia* is sensitive to both Amp and Tet [27, 38], whereas all three symbionts are affected by Tet [27]. Finally, irradiation significantly affects *Sodalis* and *Wolbachia*, but not *Wigglesworthia* titers [40]. Thus, our treatments include tsetse flies with their full microbiota (untreated controls of both antibiotic and irradiation experiments), flies with normal *Sodalis* and *Wolbachia* titers but without *Wigglesworthia* (*Gmm*^*Wgm-*^ resulting from Amp treatment), flies with normal *Wigglesworthia,* but reduced *Sodalis* and *Wolbachia* titers (*Gmm*^Sod-Wlb-^, resulting from some of the irradiation treatments) and fully aposymbiotic flies (*Gmm*^Apo^, resulting from Tet treatment). Tet treatment, which clears all symbionts, had the strongest and most consistent effect on CHC profiles of males and females, as well as on the relative amount of the female sex pheromone. Furthermore, males mate preferentially with untreated females, possibly because their CHC profile contains a higher relative amount of 15,19,23-trimethyl-heptatriacontane, the female sex pheromone. Amp treatment also affected CHC profiles in males and 5-day old females, but not in 10-day old females, while irradiation which disturbs *Sodalis* and *Wolbachia* did not affect the CHC profiles of male flies. However, we cannot exclude the possibility that other time points of irradiation would yield different results, given the complex interaction effects of irradiation dosage and time on symbiont titers [40]. Nevertheless, taken together, these results suggest that *Wigglesworthia* has the strongest effect on CHC profiles of *G. m. morsitans.*

There is increasing evidence that symbiotic bacteria can under certain circumstances influence pheromone communication and mate choice of their insect host [7, 14–16, 43–49], which can ultimately result in reproductive isolation and, hence, speciation [11, 17, 44]. While reproductive manipulators like *Wolbachia* are prime suspects for the modification of their host’s chemical communication and mate choice [11, 17, 44], several gut associated microbes are also known to be involved in the production of host pheromone components. By contrast, nutritional endosymbionts like *Wigglesworthia* were so far not implicated in changes of host mating signals or mate choice. However, as *Wigglesworthia* provides essential vitamins [24] and is involved in the maturation of the immune system [25, 26, 27] direct or indirect effects on other metabolic processes such as the synthesis and distribution of hydrocarbons or their precursors seem plausible and could explain the modification of CHC profiles upon antibiotic treatment observed in our study.

Although our results are consistent with the hypothesis that an effect of *Wigglesworthia* on CHC profiles modulates mate choice, we could not test this effect on mate choice directly, nor is it currently possible to exclude direct effects of the antibiotic treatment itself on the fly’s physiology, CHC profile, overall vigor, and behavior. Antibiotics influence several life history parameters of insects. For example, treatment of the black bean aphid (*Aphis fabae*; [48, 49])*,* the mustard aphid (*Lipaphis erysimi*; [50]), the walnut husk fly (*Rhagoletis complete*; [51]) and the melonfly (*Dacus cucurbitae*; [52]) with Tet derivatives in particular causes diverse side effects including reduced larval development rate, adult size, weight, reproduction and longevity. However, as all these aphids harbor the obligate intracellular mutualist *Buchnera aphidicola* [53], and the gut microbiota of diverse true fruit flies also has a significant influence on host fitness [54, 55], a direct influence of the tested antibiotics on host physiology cannot be differentiated from an indirect influence via symbiont depletion in these studies. A few studies have succeeded in implicating the insect microbiota in CHC profile modulation, without the involvement of antibiotics. Guo et al. [56] demonstrated that the gut microbiota of termites provides precursors for the synthesis of methyl-branched CHCs through the incorporation of ^14^C-labelled succinate. Furthermore, Dosmann et al. [57] investigated a possible microbial modulation of nest mate recognition in harvester ants by altering the external microbiome through antibiotic treatment, but also exclusively through the application of cultured bacteria to the ant cuticle. While the application of cultured microbes influenced nest mate recognition, treatment with rifampicin did not [57]. Thus, while direct contributions of obligate symbionts to nest mate recognition cues in harvester ants are possible, the results remain inconclusive.

Another factor that warrants careful interpretation of the presented results is that differences in CHC profiles across experiments were more pronounced than between treatments within each experiment (Additional file 1). Hence, the age of the flies, the rearing conditions (individual vs. mass-rearing), and possibly fluctuations in rearing conditions (e.g. diet, temperature, humidity) as well as variation in the genetic composition of the starting populations may influence CHC composition. Furthermore, the *Sodalis* and *Wolbachia* depleted flies resulting from gamma-irradiation were treated themselves, as opposed to analyzing the offspring of treated flies, as in the antibiotic experiments. Flies thus have experienced a different ontogeny, with *Sodalis* and *Wolbachia* present during part of their development. Despite the fact that cuticular hydrocarbons usually display a fast turnover, enabling insects to adapt within hours to days, it cannot be excluded that the late time point of symbiont depletion in the irradiation treatment was responsible for the lack of an effect on CHC profiles. Age and ontogeny-dependent changes in CHCs have been described across different insect species [58–62] and may serve as reliable age indicators for mate choice [63]. Diet and host genetics influence CHCs via fatty acid metabolism [64, 65], whereas fluctuations in temperature and humidity can stimulate insects to adjust their CHC profiles to improve desiccation resistance under the current conditions [66–68]. Thus, under natural settings, microbial symbionts may be one of several different factors affecting insect CHC profiles and thereby mate choice and sexual selection.

## Conclusion

Our results provide first insights into changes in CHC profiles upon symbiont depletion by antibiotic and gamma-irradiation treatment in *G. m. morsitans*. Individual rearing corroborated the results obtained from mass-rearing, excluding potential pseudoreplication artifacts by flies exchanging CHCs through direct contact under mass-rearing conditions. Mate choice assays indicate that antibiotic treatment not only affects CHC composition, but also impairs mating success of both males and females. However, the link between mating success, CHC profiles, and *Wigglesworthia* as the causative agent for the observed changes remains speculative at this point. Further studies are needed to pinpoint single symbiont contributions to CHC synthesis and mate choice. Nevertheless, our results indicate that the chemical ecology of tsetse flies should be taken into account when investigating the effects of symbionts on host fitness or manipulating the symbiosis to enhance refractoriness to trypanosome infection. Furthermore, we could show that gamma-irradiation, which is routinely employed to create sterile males for the sterile-insect-technique to control *G. m. morsitans* populations, does not alter the CHC profiles of males. Hence, irradiated males might not suffer a competitive disadvantage after their release into the field, if females use chemical cues for mate choice. Finally, if symbiont or parasite infection predictably affects CHC profiles, chemical analyses may also provide a simple and cost-efficient alternative to molecular screenings for the assessment of symbiont/parasite infection status.

## Additional files

Additional file 1: Comparison of CHC profiles of untreated (Ctr) and tetracycline-treated (Tet) (**a**) female and (**b**) male tsetse flies (*G. m. morsitans*) across the three different experiments. (PDF 1363 kb)

Additional file 2: Effect of gamma-irradiation dose and time point on CHC profiles of 10 day old individually reared adult *G. m. morsitans* males. Discriminant analysis based on log-ratio transformed relative amounts across all treatment groups (time points and irradiation doses). (PDF 1353 kb)

Additional file 3: Effect of the time point of gamma-irradiation on CHC profiles of individually reared 10 day old adult *G. m. morsitans* males. Discriminant analysis based on log-ratio transformed relative amounts across irradiation time points (early and late pupal development and as young adults). (PDF 1354 kb)

## Abbreviations

Amp: Ampicillin
ANOVA: Analysis of variance
Apo: Aposymbiotic
CHC: Cuticular hydrocarbon
DA: Discriminant analysis
FAO/IAEA: Joint food and agriculture organization and international atomic energy agency of the United Nations
Gmm: *Glossina morsitans morsitans*
Tet: Tetracyclin
Wgm: *Wigglesworthia* depleted
