# Supplementary material for: Effect of antibiotic treatment and gamma-irradiation on cuticular hydrocarbon profiles and mate choice in tsetse flies (Glossina m. morsitans)
Source: BMC Microbiol. 2018 Nov 23;18(Suppl 1):145. doi: 10.1186/s12866-018-1292-7 (PMC6251160; doi:10.1186/s12866-018-1292-7)
Supplement: Supplementary file 1 — Comparison of CHC profiles of untreated (Ctr) and tetracycline-treated (Tet) (a) female and (b) male tsetse flies (G. m. morsitans) across the three different experiments. (PDF 1363 kb) [file 12866_2018_1292_MOESM1_ESM.pdf]

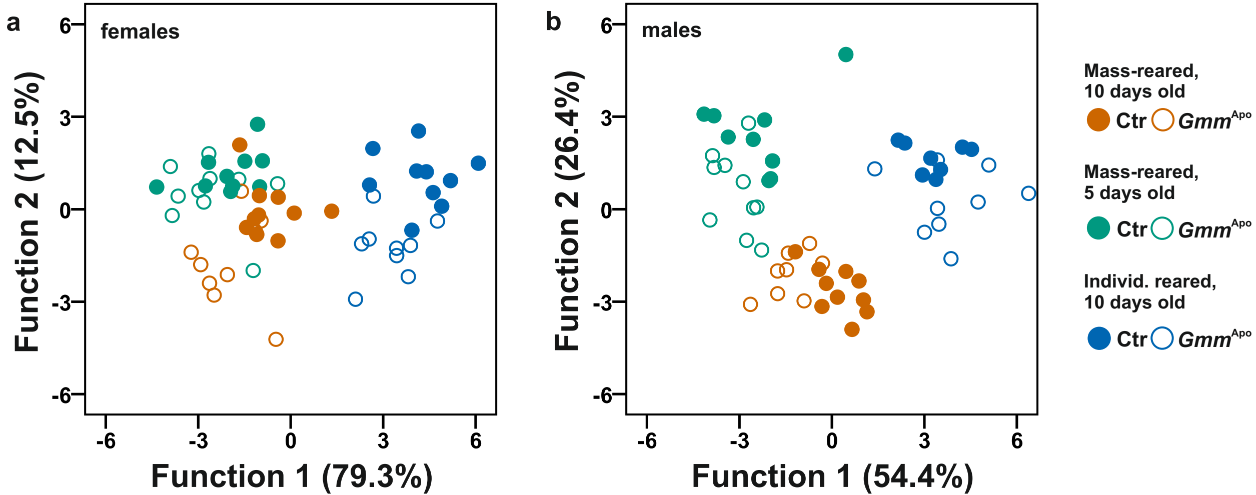

a
females
Function 2 (12.5%)
Function 1 (79.3%)
6
3
0
-3
-6
b
males
Function 2 (26.4%)
Function 1 (54.4%)
Mass-reared, 10 days old
Ctr GmmApo
Mass-reared, 5 days old
Ctr GmmApo
Individ. reared, 10 days old
Ctr GmmApo
