# Supplementary figures and images for: Effect of antibiotic treatment and gamma-irradiation on cuticular hydrocarbon profiles and mate choice in tsetse flies (Glossina m. morsitans)
Source: BMC Microbiol. 2018 Nov 23;18(Suppl 1):145. doi: 10.1186/s12866-018-1292-7 (PMC6251160; doi:10.1186/s12866-018-1292-7)

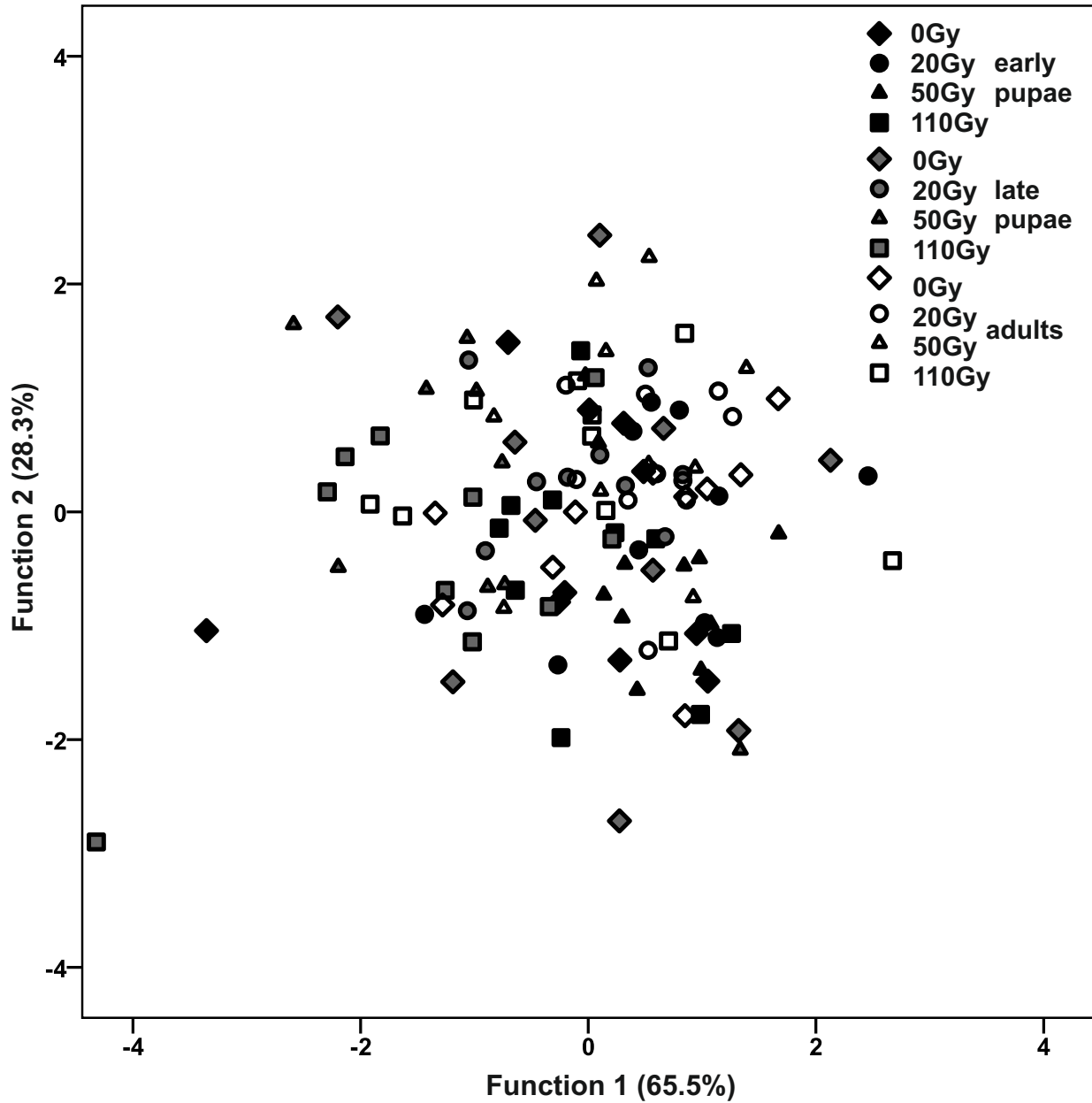

Supplement: Supplementary file 2 — Effect of gamma-irradiation dose and time point on CHC profiles of 10 day old individually reared adult G. m. morsitans males. Discriminant analysis based on log-ratio transformed relative amounts across all treatment groups (time points and irradiation doses). (PDF 1353 kb) [file 12866_2018_1292_MOESM2_ESM.pdf]

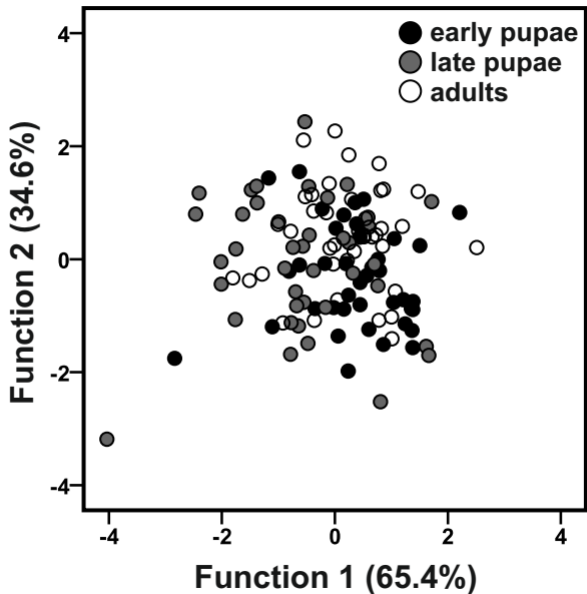

Supplement: Supplementary file 3 — Effect of the time point of gamma-irradiation on CHC profiles of individually reared 10 day old adult G. m. morsitans males. Discriminant analysis based on log-ratio transformed relative amounts across irradiation time points (early and late pupal development and as young adults). (PDF 1354 kb) [file 12866_2018_1292_MOESM3_ESM.pdf]
